# Formulation and Evaluation of Exotic Fat Based Cosmeceuticals for Skin Repair

**DOI:** 10.4103/0250-474X.44615

**Published:** 2008

**Authors:** S. D. Mandawgade, Vandana B. Patravale

**Affiliations:** Department of Pharmaceutical Sciences and Technology, University Institute of Chemical Technology (UICT), Mumbai-400 019, India

**Keywords:** Mango butter, foot care cream, wound healing, skin repair

## Abstract

Mango butter was explored as a functional, natural supplement and active skin ingredient in skin care formulations. A foot care cream was developed with mango butter to evaluate its medicinal value and protective function in skin repair. Qualitative comparison and clinical case studies of the product were carried out. Wound healing potential of foot care cream was investigated on the rat excision and incision wound models. Results of the clinical studies demonstrated complete repair of worn and cracked skin in all the human volunteers. Furthermore, foot care cream exhibited significant healing response in both the wound models. The project work could be concluded as establishment of high potential for mango butter to yield excellent emolliency for better skin protection. Improving the product features and medicinal functionality further validate mango butter as a specialty excipient in development of cosmeceuticals and has an immense value for its commercialization.

In daily life, human skin is exposed to variety of environmental factors and unsanitary conditions that have detrimental effects on dermal integrity. The consequences are dry skin, wrinkles, cracks and eventually sepsis. The most protective and preventive step taken against dry skin formation and related disorders is the use of “emollients” or moisturizing creams and lotions preferably with antiseptic properties. Exotic lipids/fats obtained from de-shelled, pressed fruit kernels of shea, sal, mango and kokum tree are beneficial ingredients and have many attributes that make them suitable for skin care. This popularity has even increased in recent years; a result of fats not only being an excellent emollient, but also offering interesting bioactive properties. Furthermore, these natural lipids have high oxidative resistance and are biocompatible, having no deleterious effects on the skin. The exotic fats used in skin care are known to exhibit their effects through restoration of a sufficient layer of skin lipids and skin elasticity, boost natural skin regeneration and increased skin hydration by forming an inert, epicutaneous occlusive membrane. Besides these facts, mango butter which is one of several exotic fats is viewed as an ingenious replacement for cocoa butter, mineral and petroleum based emollients because of its appreciable contents which are very important as source of skin active ingredients. Literature survey on principle constituents of mango butter very well reveals its bacteriostatic, antimicrobial activity and anti-inflammatory activity^1^, signifying its supplemental use as curative and protective medicine in topical formulations. The high percentage of tocopherols, phytosterols and triterpenes in the mango butter significantly reduce wrinkles and roughness of the skin while the repairing and protecting properties open up possibilities to formulate caring products for sensitive skin.

The aim of this study was to evaluate mango butter as a functional, natural supplement and skin active ingredient, hitherto unexplored. Further objective was to develop foot care cream (FCC) to explore the medicinal value and commercial application of mango butter in skin care cosmetic formulary.

Mango butter and refined fractions thereof were the kind gift samples from Charbhuja Trading Agencies and Pvt. Ltd. Mumbai, India. All other ingredients of extra pure grade were purchased from local market and used as such.

Foot care cream was formulated with mango butter and olein fraction thereof as a base (25%w/w), fortified with vitamin E acetate (1%w/w). The product was oil-in-water emulsion system which was stabilized by emulsifiers and proper concentration of preservative. The oil phase comprised of emulsifiers, emulsion stabilizers, mango butter and olein fraction, replacing the mineral and petroleum emollients from conventional formulas whereas; aqueous phase comprised of water-miscible additives, preservative and double distilled water. Both the phases were separately heated to 65-70° and then aqueous phase was incorporated into oil phase with homogenization whilst maintaining the temperature for 2-3 min. Stirring was continued with gradually lowering of the temperature. Perfume was added to the preparation at 40-45° and stirred well to yield the product. The pre-formulation studies and product optimization was carried out with respect to quantity of mango butter and olein fraction to be incorporated for superior emolliency, percentage of emulsifiers, percentage of emulsion stabilizers, percentage of essential additives (humectant, vitamin E acetate, preservative), percentage of an aqueous phase and aesthetics. The production batches were simultaneously evaluated and optimized for appearance, emulsion stability, spreadability, skin feel, smoothness and absorption. The stability of the developed FCC was studied at 10±2°, 45±5° and at room temperature for 1 y.

Draize repeated insult patch test (DRIPT) was performed for FCC on New Zealand rabbits weighing 2.5-3 kg of either sex. The study protocol was approved by the Institutional Animal Ethical Committee (Approval No. UICT/PH/IAEC/0405/7). FCC was applied once daily for seven days on the hair free skin of rabbits by uniformly spreading 0.5 g within the area of 4 cm^2^. The skin was observed for any visible change such as erythema (redness) or oedema (swelling) after every 24 h for seven days. Evaluation was done by using the scale given by Draize.

Eight weeks clinical studies were carried out on six healthy human volunteers suffering from different foot ailments. The efficacy of product was judged on functionality attributes (reduction in amplitude of cracked heels, pain and bleeding through the cracks, degree of healing, skin re-construction, soothing, skin rehydration and as an antiseptic against the growth of resident microorganisms) and aesthetic attributes (appearance, spreadability, skin feel, smoothness and absorption). Any adverse reaction on skin towards ingredients used was also examined during the study. The visible effects were observed weekly and noted as a product evaluation report.

Wistar rats were divided into three groups (control, test and marketed) each containing six animals. They were housed individually in the animal house with food and water given *ad libitum*. The study protocol was approved by the Institutional Animal Ethical Committee (Approval No. UICT/PH/IAEC/0905/28). The animals were depilated at the desired site before creating the wound. FCC was applied topically once a day throughout the study period.

Excision wound was produced in rats as per the method described by Morton and Malone^4^ under light ether anesthesia. The skin of the impressed area was excised to full thickness to obtain a wound area of about 500 mm^2^. The parameters studied were percent wound closure, re-epithelization time and scar area. The percent wound closure was recorded on d 4, 8 and 16. The scar shape and area were traced and measured planimetrically. Resutured Incision wound method was performed as described by Ehrlich and Hunt^5^. On the 8th post wounding day, sutures were removed and the breaking strength was determined on 10th post wounding day by continuous constant water flow technique of Lee^6^.

The results from wound model studies were expressed as mean±SE. The significance of differences between the means was analyzed by student’s *t*-test followed by Tukey’s test. A probability of p<0.05 was considered significant.

An O/W emulsion system containing 25%w/w of mango butter and its olein fraction and 60%w/w aqueous phase could be successfully, reproducibly formulated to yield foot care cream with excellent emolliency. The developed formulation was non-sticky, non-oily and easily water washable. In comparison to the marketed product, developed FCC scored better with respect to appearance, spreadability, smoothness, skin feel and absorption. The stability studies ascertained good product shelf-life with respect to pH, emulsion stability, microbial resistance, color, fragrance and viscosity over the time period tested.

Primary skin irritation studies for FCC showed no skin irritation (primary skin irritation index = 0) in consecutive applications for seven days on intact rabbit skin. This indicates better skin acceptability of the developed formulation for topical application.

Clinical studies showed no irritancy, sensitivity towards the skin in all the human volunteers and the results were highly significant with the developed product at the end of study. Complete repair of cracked skin was seen in all the volunteers (fig. 1). Its antiseptic, healing, soothing and cooling actions were predominant in most of the clinical subjects, unlike organic oils. Excellent emolliency in the formulation rebuilt a naturally occlusive, protective skin barrier and actively replenished moisture, leaving the skin looking silky smooth and hydrated. It had the best appearance, spreadability, skin feel, smoothness and absorption in comparison. The comparative evaluation of product efficacy with respect to its functional and aesthetic attributes is illustrated in figs. 2 and 3, respectively. The marked skin regeneration and restoration function of FCC in clinical studies on healthy human volunteers was authenticated by studying *in vivo* wound repair in excision and incision wound models in the Wistar rats. To evaluate efficacy *in vivo*, percent wound closure, time of re-epithelization and size of scar area in excision model and tensile strength of wound in incision was studied.

**Fig. 1:**
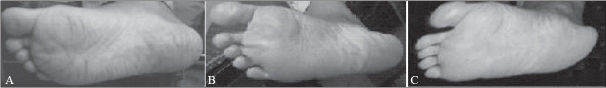
Photographs showing clinical efficacy of foot care cream. A, B and C represent clinical conditions on 0 d, after 4 w and after 8 w period, respectively.

**Fig. 2:**
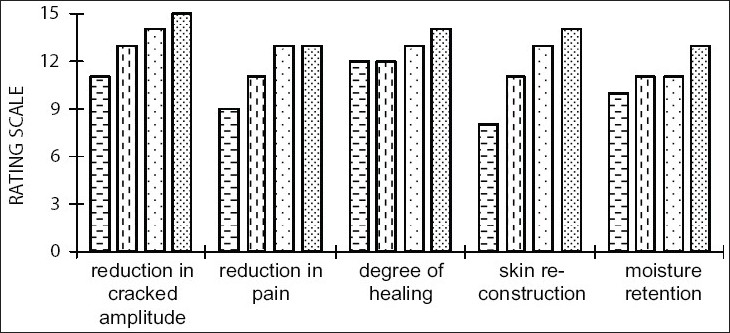
Efficacy of foot care cream with respect to its functional attributes. Evaluation of efficacy of foot care cream with respect to its functional attributes. (Scale values are average of rating scores from six volunteers). Efficacy of foot care cream at the end of 2 w 
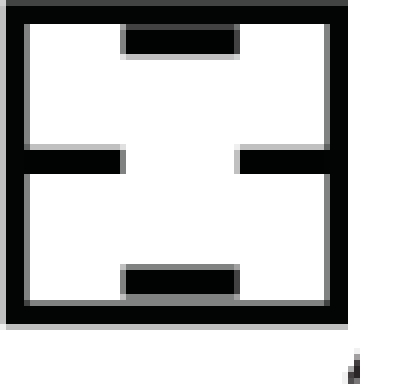
, 4 w 
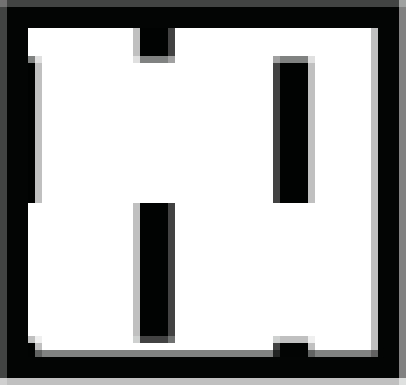
, 6 w
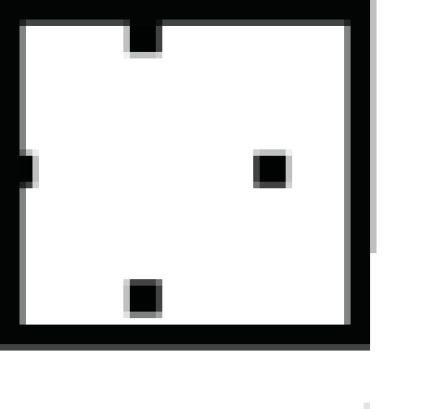
, 8 w 
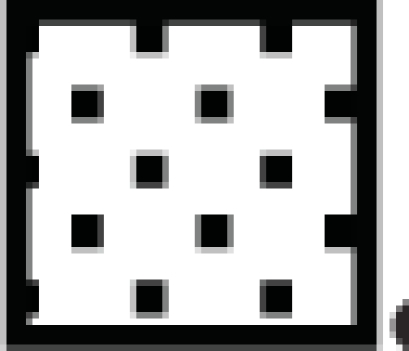
.

**Fig. 3:**
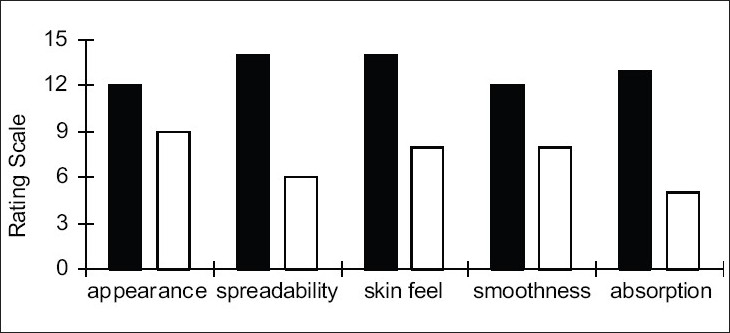
Comparative evaluation of efficacy of foot care cream and the aesthetic attributes. Comparative evaluation of efficacy of foot care cream with respect to its aesthetic attributes. (Scale values are average of rating from a panel of pharmacists). Efficacy of (■) foot care cream and (□) marketed cream with respect to aesthetic attributes.

When compared to control (78.1±1.6%) on the d 16, FCC (91.1±1.2%) showed significantly better wound closure while a similar improvement in wound closure was seen in marketed (povidone iodine ointment, 85.5±0.7%) group but when compared amongst these groups, results of FCC were superior (p<0.05) to marketed group. This evidently supports the healing effects and reduction in crack amplitude in the clinical studies. Complete re-epithelization in 16.8±0.6 and 19.2±0.5 d was seen against control (24±0.7 d) at the end of study for FCC and marketed group, respectively. It signifies better skin regeneration and thereby its repair (decreased time of re-epithelization) (p<0.05). Further, in the excision model, the results indicate the least scar areas for FCC group (1.05±0.23 mm^2^) followed by marketed group (1.08±0.04 mm^2^). These were significantly decreased (p<0.05) when compared to control group with scar area of 1.38±0.05 mm^2^. Such minimal scar formation and leaving the skin free of marks by treatment with FCC on skin ruptures and lesions is remarkable from the perspective of cosmeceuticals.

The tensile strength in incision wound model was evaluated by continuous constant water flow technique. FCC and marketed group showed increased mean tensile strength, compared to control (160.0±8.6 g). The maximum tensile strength was seen in group treated with FCC (341.7±23.1 g, p<0.001) followed by marketed (215.0±6.2 g, p<0.05) which were statistically significant from control. All the results are summarized in Table 1.

**TABLE 1:** COMPARATIVE EVALUATION OF THE PARAMETERS IN *IN VIVO* EXCISION AND INCISION WOUND MODELS

Group	Excision					Incision
		
	Percentage Wound Contraction			Time of Re-epithelization (d)	Scar Area (mm^2^)	Tensile Strength (g)
				
	d 4	d 8	d 16			
Control	13.2±3.6	50.9±4.9	78.1±1.6	24.0±0.7	1.38±0.05	160.0±8.6
Foot Care Cream	14.4±3.1	41.9±7.1	91.1±1.2^#^	16.8±0.6^#^	1.05±0.23*	341.7±23.1^#^
Marketed Cream	17.3±3.5	51.1±4.4	85.5±0.7*	19.2±0.5^#^	1.08±0.04*	215.0±6.2*

All values are given in mean±SE

* p<0.05 = significant *Vs*. Control.

Overall, mango butter has a high potential to yield excellent emolliency which rebuilds a naturally occlusive, protective skin barrier and actively replenishes moisture for better skin protection thereby leaving the skin silky, smooth and hydrated. The repairing and protecting properties open up possibilities to formulate caring products for sensitive skin. Improving the product features and medicinal functionality further validate mango butter as a specialty excipient in development of cosmeceuticals and has an immense value for its commercialization.
